# Inhibition of Glycogen Synthase Kinase-3β Counteracts Ligand-Independent Activity of the Androgen Receptor in Castration Resistant Prostate Cancer

**DOI:** 10.1371/journal.pone.0025341

**Published:** 2011-09-29

**Authors:** Stefanie V. Schütz, Andres J. Schrader, Friedemann Zengerling, Felicitas Genze, Marcus V. Cronauer, Mark Schrader

**Affiliations:** Department of Urology, Ulm University, Ulm, Germany; University of Missouri-Columbia, United States of America

## Abstract

In order to generate genomic signals, the androgen receptor (AR) has to be transported into the nucleus upon androgenic stimuli. However, there is evidence from *in vitro* experiments that in castration-resistant prostate cancer (CRPC) cells the AR is able to translocate into the nucleus in a ligand-independent manner. The recent finding that inhibition of the glycogen-synthase-kinase 3β (GSK-3β) induces a rapid nuclear export of the AR in androgen-stimulated prostate cancer cells prompted us to analyze the effects of a GSK-3β inhibition in the castration-resistant LNCaP sublines C4-2 and LNCaP-SSR. Both cell lines exhibit high levels of nuclear AR in the absence of androgenic stimuli. Exposure of these cells to the maleimide SB216763, a potent GSK-3β inhibitor, resulted in a rapid nuclear export of the AR even under androgen-deprived conditions. Moreover, the ability of C4-2 and LNCaP-SSR cells to grow in the absence of androgens was diminished after pharmacological inhibition of GSK-3β *in vitro*. The ability of SB216763 to modulate AR signalling and function in CRPC *in vivo* was additionally demonstrated in a modified chick chorioallantoic membrane xenograft assay after systemic delivery of SB216763. Our data suggest that inhibition of GSK-3β helps target the AR for export from the nucleus thereby diminishing the effects of mislocated AR in CRPC cells. Therefore, inhibition of GSK-3β could be an interesting new strategy for the treatment of CRPC.

## Introduction

Transcriptional regulation by nuclear receptors plays a pivotal role in the development and growth of prostate cancer (PC) cells. This is well exemplified by the role of the androgen receptor (AR), a ligand-activated transcription factor belonging to the steroid receptor superfamily. In the absence of androgens, the AR is predominantly located in the cytoplasm stabilized by a multichaperone complex [1]. Upon binding of androgens, the AR is thought to undergo a conformational change leading to a homodimerization with another AR protein after dissociation from parts of the multichaperone-complex [2]. Subsequently, the AR is actively transported into the nucleus where it binds to specific DNA-sequences termed androgen response elements (ARE) found within promoter or enhancer regions of AR target genes [3] thereby activating the general transcription apparatus [4].

The initial androgen dependency of prostate cancer cells is the reason why most PC respond to androgen ablation therapy. Unfortunately, the benefit of androgen ablation is only transitory. After a period of around two years, PC almost invariably progress to a state of the disease termed castration-resistant prostate cancer (CRPC) where tumor cells can grow and survive under castrate levels of circulating androgens. Although the *in vitro* development of an androgen-independent phenotype is mostly based on the loss of the AR in tumor cells, there is evidence from several clinical studies that the AR is rarely lost in CRPC cells *in vivo* [5], [6]. Furthermore, these studies suggest that resistance to conventional hormonal therapy is not due to a loss of androgen sensitivity but rather may be a consequence of a deregulated AR-signalling axis [7].

In order to generate genomic signals in hormone-dependent PC cells the AR must be transported into the nucleus upon androgenic stimuli. There is compelling evidence from *in vitro* experiments that in a subset of CRPC cells, the AR acquires the ability to undergo ligand-independent shuttling from the cytoplasm to the nucleus [8]. Most interestingly, in these cells the AR displays a high level of constitutive, androgen-independent activity [9]. As the nuclear presence of the receptor is a prerequisite for AR signalling, regulation of nuclear translocation might reveal new strategies for the treatment of both androgen-dependent PC as well as CRPC.

Various studies have offered insights into the nuclear import of the AR. A bipartite nuclear localization sequence (NLS) has been identified in the DNA binding domain (DBD) and hinge region of the AR [10]. This NLS utilizes the classical importin pathway for transport through the nuclear pore complex [11], [12]. In addition, a less defined NLS is present in the ligand binding domain (LBD) of the AR [13], [14]. In contrast to the nuclear import of the AR, the mechanisms involved in AR nuclear export are poorly understood. The DNA binding domains (DBD) of diverse steroid receptors have been suggested to function as nuclear export signals (NES) for a calreticulin mediated nuclear export [15]. However, for the AR these data have been discussed controversially [16], [17]. Recently, Saporita et al. reported the identification of a novel NES (amino acids 743–817) situated in the LBD of the AR [18]. The identification of a NES in the LBD of the AR is in agreement with previous findings of our group studying the nuclear export of the AR in androgen-stimulated PC cells after inhibition of the glycogen-synthase-kinase 3β (GSK-3β) [19].

The finding that inhibition of GSK-3β induces a rapid nuclear export of the AR in androgen-stimulated PC cells prompted us to analyze the effects of GSK-3β inhibition in castration-resistant LNCaP sublines C4-2 and LNCaP-SSR [20], [21], both known to exhibit high levels of nuclear AR in the absence of androgenic stimuli. Both *in vitro* as well as *in vivo* data presented in this study suggest that induction of nucleocytoplasmic AR export might be a useful strategy to diminish AR signalling, especially in advanced CRPC.

## Results

### AR and GSK-3β are up-regulated in CRPC cell lines

**Figure 1:**
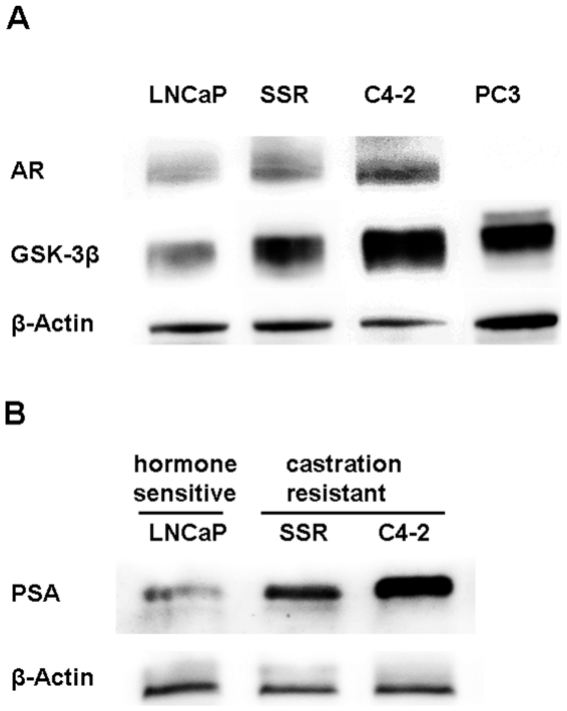
Intracellular levels of AR, GSK-3β and PSA in different PC cell lines. (A) Intracellular AR and GSK-3β-protein levels in PC cell lines grown in the absence of androgens. The AR-positive LNCaP, LNCaP-SSR and C4-2 cells as well as the AR-negative PC3 cells were grown for 48 hours under androgen-deprived conditions. Subsequently, cells were lysed and cell lysates were analyzed by Western blotting for AR and GSK-3β. β-actin bands served as loading control. (B) Intracellular PSA levels in CPRC cells grown under androgen-deprived conditions. AR-positive LNCaP, LNCaP-SSR and C4-2 cells were grown and treated as described. Relative PSA levels were determined in the corresponding cell lysates by Western blotting as described in Material and Methods. β-actin bands served as loading control.

In order to analyze the effects of GSK-3β inhibitors on AR-activity in CRPC cells we first determined endogenous AR or GSK-3β levels in whole cell extracts of PC cells grown in the absence of DHT. The AR-positive LNCaP sublines C4-2 and LNCaP-SSR, known to proliferate in the absence of androgens, served as models for CRPC [20], [21]. The androgen-sensitive LNCaP as well as the AR-negative PC3 cells served as controls. As seen in Figure 1A, the AR was detectable in all LNCaP lines grown in the absence of the AR-stabilizing androgen DHT. Intracellular AR levels were significantly increased in the AR-positive CRPC cells (C4-2 +177%, LNCaP-SSR +126%) (Table 1). The increase of AR-protein in castration-resistant LNCaP sublines was paralleled by an increase in PSA (C4-2 +337% and LNCaP-SSR +110%, respectively), the latter suggesting that the AR is particularly active in these cells (Figure 1A, B; Table 1). Western blot analysis of intracellular GSK-3β protein revealed significantly higher intracellular GSK-3β levels in C4-2 (+361%) and LNCaP-SSR (+150%) cells in comparison to the parental LNCaP cells (Figure 1A, Table 1).

**Table 1:** Intracellular levels of AR, PSA and GSK-3β in different prostate cancer cell lines.

	LNCaP	LNCaP-SSR	LNCaP C4-2	PC3
**% AR/actin**	100±7	226±11 *	377±33 *	2±1
**% PSA/actin**	100±10	210±4 **	437±54 ***	nd
**% GSK-3β/actin**	100±25	250±35 #	461±18 ##	192±19

Western blots were performed as described under Figure 1. Protein levels of AR, PSA, GSK-3β were quantified by densitometry and normalized to the corresponding actin levels (loading control). Results are expressed in relation to the corresponding LNCaP controls which were set at 100%. Each value represents the mean of 3 independent experiments ± standard deviation (p-values compared to corresponding LNCaP values:

*p = 0.006;

**p = 0.002;

***p = 0.006;

# p = 0.016;

## p<0.002. nd  =  not determined).

### The maleimide SB216763 reduces GSK-3β-activating phosphorylation at tyrosine 216

**Figure 2:**
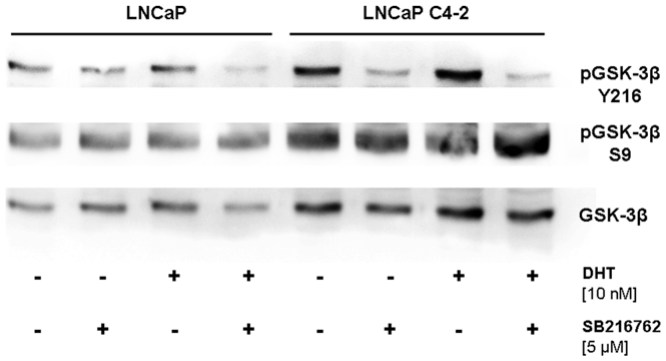
SB216763 inhibits phosphorylation of GSK-3β at tyrosine 216. LNCaP cells and C4-2 cells were seeded into T25 flasks and allowed to adhere overnight. After that, medium was replaced by steroid-free medium and cells were grown for another 24 hours. Subsequently, SB216763 (final concentration 5 µM) was added 30 min prior to DHT (10 nM) treatment. After 4 hours, cell lysates were analyzed for GSK-3β^Y216^ and GSK-3β^S9^ phosphorylation as described in Material and Methods.

Several studies reported that the majority of the GSK-3β molecules are inactive in LNCaP cells as a result of a phosphorylation at serine 9 (S9) [26], [27]. However, there is experimental evidence that GSK-3β activity it is not fully inhibited in LNCaP cells [28], [29], [30], [31], [32]. Following DHT treatment, phosphorylation of GSK-3β at tyrosine 216 (GSK-3β^Y216^), a GSK-3β-activating phosphorylation site, was increased in LNCaP and C4-2 cells (Fig. 2). In order to inhibit residual GSK-3β activity in LNCaP and C4-2 cells, cells were treated with the maleimide SB216763, a GSK-3β inhibitor recently shown to inhibit the phosphorylation of GSK-3β^Y216^ [33]. Furthermore, the ability of SB216763 to inhibit GSK-3β activity in C4-2 cells was verified by alterations in the phosphorylation status of β-catenin, a prototypical downstream target of GSK-3β (Figure S1).

As expected, incubation with SB216763 inhibited the phosphorylation of GSK-3β^Y216^ in both cell lines, being most pronounced in the androgen-independent C4-2 cells (Fig. 2). Most interestingly, an SB216763 induced decrease of GSK-3^Y216^ phosphorylation in DHT-treated C4-2 cells was paralleled by an increase in S9 phosphorylation (Fig. 2).

### Unliganded AR is localized to the nucleus in CRPC LNCaP-sublines

**Figure 3:**
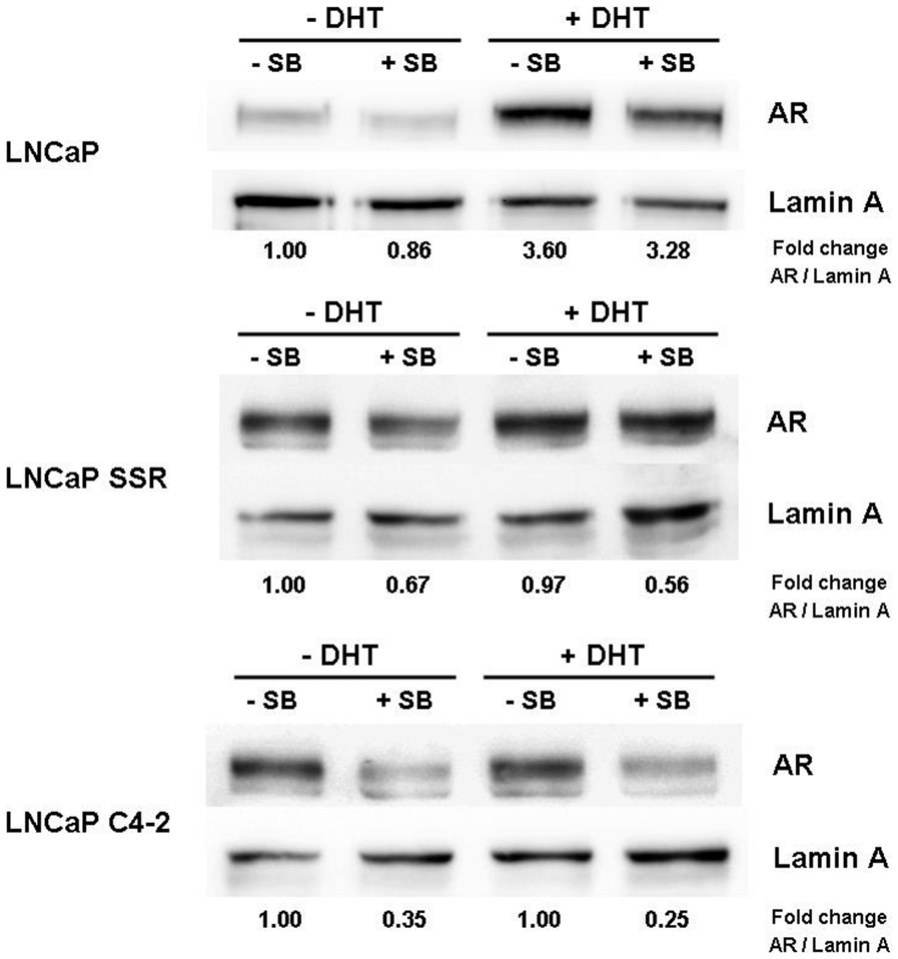
Inhibition of GSK-3β by SB216763 enhances nuclear export of the AR. Cells were grown under steroid-free conditions for 24 hours. Cells were then treated with 10 nM DHT and incubated for another 30 min. Subsequently, SB216763 was added (end concentration 5 µM) and cells were allowed to grow for another 210 min. Following this treatment, nuclear extracts were prepared and analyzed for AR and lamin A as described in Material and Methods. AR and lamin A levels were quantified by densitometry. AR levels were expressed in fold-change AR/Lamin of cells grown in absence of DHT and SB216763, which was set at 1.

**Figure 4:**
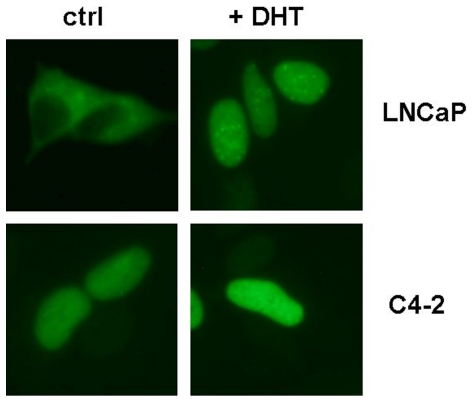
Effect of SB216763 on the localization of unliganded AR-Eos fusion protein in LNCaP and C4-2 cells. LNCaP cells and C4-2 cells were transfected with pAR-t1EosFP and grown for 24 hours in the absence of androgens. Thereafter, cells were treated with/without DHT (final concentration 10 nM). After 4 hours nuclear or cytoplasmic localisation of AR-EosFP was monitored by fluorescence microscopy.

Nuclear localization of the AR is a prerequisite for genomic signalling. As seen in Figure 3, the CRPC sublines LNCaP-SSR and C4-2 exhibit high nuclear AR in the absence of androgenic stimuli. When treated with DHT, nuclear AR was dramatically increased in the hormone-dependent LNCaP cells, whereas the increase in nuclear AR in LNCaP-SSR and C4-2 remained marginal (Fig. 3, Table 2). To further analyze the ligand-independent nuclear import of the AR in LNCaP and castration-resistant C4-2, cells were transfected with an expression construct coding for the green fluorescent AR wild type-Eos fusion protein (AR-EosFP) [19]. In contrast to LNCaP cells in which AR-EosFP was only nuclear in the presence of DHT, AR-EosFP was detectable in the nuclei of C4-2 cells in absence of androgens (Fig. 4). This finding suggests that the predominant nuclear localization of the AR in C4-2 cells is not due to an autonomous function of the receptor molecule but depends on a deregulation of cellular factors in C4-2 cells.

**Table 2:** Effects of SB216763 on the nuclear export of the AR.

	untreated controls	SB21673	DHT	DHT + SB216763
**LNCaP**	100±5	83±8 *	318±40	296±32#
**LNCaP-SSR**	100±7	59±11 **	100±4	54±13 ##
**LNCaP C4-2**	100±11	36±8 ***	113±9	28±8 ###

Western blots were performed as described under Figure 3. Nuclear AR levels were determined by densitometry normalized to the corresponding lamin levels (loading control). Nuclear AR levels are expressed in AR/lamin of corresponding controls grown in absence of DHT and SB216763 ( = 100%). Each value represents the mean of 3 independent experiments ± standard deviation (p-values compared to corresponding untreated controls:

*p = 0.016;

**p = 0.009;

***p = 0.001; p-values compared to corresponding DHT treated controls:

# p = 0.055;

## p = 0.019;

### p = 0.009).

### Treatment of castration-resistant LNCaP sublines with SB216763 triggers a CRM1-mediated nuclear export of the AR in the absence of androgens

**Figure 5:**
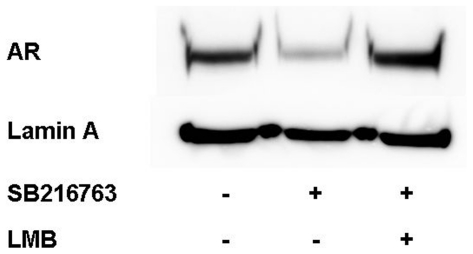
Treatment with leptomycin B reverses nuclear export of the AR induced by SB216763 in C4-2 cells. C4-2 cells grown in the absence of androgens were incubated with/without the CRM1 inhibitor leptomycin B (LMB) 30 min prior to SB216763 treatment. After 4 hours, nuclear extracts were prepared and analyzed for AR and lamin A as described in Material and Methods.

In androgen-stimulated PC cells, short term inhibition of GSK-3β activity with various small molecule inhibitors was shown to induce a rapid CRM1-dependent nucleocytoplasmic shuttling of the AR. The nuclear export was independent of the mode of action of the inhibitor used in different studies [32], [19]. In order to analyze the effects of a GSK-3β-inhibition on the androgen-independent nuclear accumulation of the AR, CRPC cell lines LNCaP-SSR and C4-2 were treated with the GSK-3β inhibitor SB216763. Parental androgen-dependent LNCaP cells served as controls (Figure 3). Treatment of C4-2 cells with SB216763 induced a rapid nuclear export of the AR in the absence/presence of androgens as shown by densitometry of three independent Western blots (AR-export rate in absence of DHT  =  64%; in presence of DHT  =  75%) (Table 2). Androgen-independent nuclear accumulation of the AR in the castration-resistant LNCaP-SSR subline was also diminished following SB216763 treatment: however, the effects were less pronounced than in C4-2 cells (41% in absence of DHT; 46% in presence of DHT) (Table 2). The nuclear export of the AR in LNCaP cells grown in the absence of androgens remained marginal but was still detectable (17% in absence of DHT; 7% in presence of DHT) (Table 3). Interestingly, the nuclear export of the AR protein in C4-2 cells grown in the absence of DHT after SB216763 treatment could be inhibited by leptomycin B confirming a CRM1-dependent export mechanism (Fig. 5).

**Table 3:** Effect of SB216763 on the nuclear localization and function of the AR *in vivo*.

	LNCaP		C4-2	
	controls	SB216763	controls	SB216763
**% nuclear AR**	74.6±25.8%	76.4±18%	63.4±10%	27.1±19.9%
**% PSA**	3.1±3,3%	0.9±0,9%	16.8±10.6%	3.8±4.7%

The CAM assay was performed with C4-2 cells as described in Material and Methods. Mean percentage ± standard deviation of AR-positive nuclei or PSA-positive cells were determined by 3 independent technicians counting 3–5 fields in 3–6 eggs.

### Silencing and long term inhibition of GSK-3β in C4-2 cells

**Figure 6:**
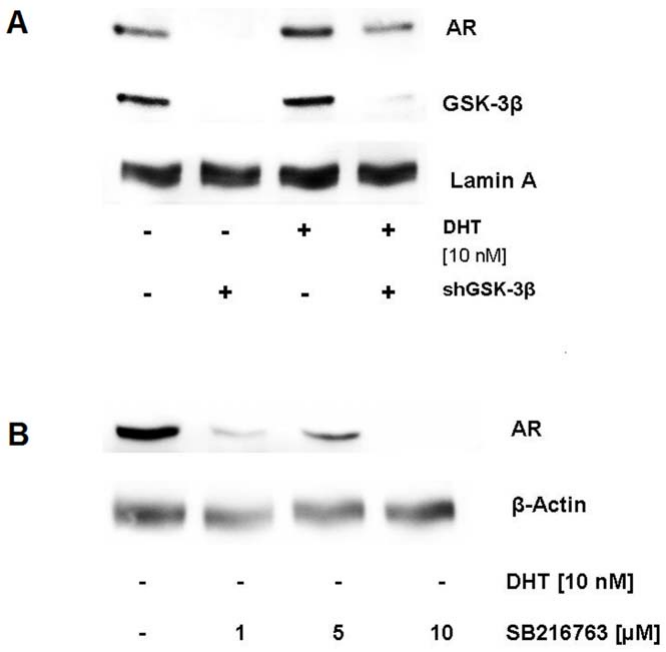
Long term inhibition of GSK-3β in C4-2 cells. (A) Silencing of GSK-3β in C4-2 cells. C4-2 cells were transiently transfected with pKD-GSK-3β-v1 or pKD-NegCon-v1. 48 hours after transfection, 10 nM DHT was added where indicated. After another 24 hours, nuclear extracts were prepared as described in Material and Methods. (B) Long term inhibition of GSK-3β using SB216763. AR-positive C4-2 cells were treated with increasing amounts of SB216763 for 48 hours in the absence of androgens. Cells were lysed and cell lysates were analyzed by Western blotting for AR. β-actin bands served as loading control.

Using a commercial validated shRNA directed against GSK-3β (32), we were able to dramatically reduce intracellular GSK-3β levels in C4-2 cells (Fig. 6A). This down-regulation was paralleled by a depletion of nuclear AR protein in C4-2 cells. Nuclear depletion of AR protein was less pronounced when C4-2 cells were grown in the presence of the AR-stabilizing hormone DHT (Fig. 6A). Long term treatment of androgen-stimulated LNCaP and 22Rv1 cells with different pharmacological GSK-3β inhibitors has repeatedly shown that GSK-3β is involved in AR stability [27], [32]. The fact that C4-2 cells express high levels of cytoplasmic as well as nuclear AR in the absence of androgens prompted us to analyze the effects of SB216763 on intracellular AR levels. As seen in Fig. 6B, long term treatment with SB216763 leads to a down-regulation of the AR protein in C4-2 cells grown in the absence of androgens.

### AR-positive CRPC cells C4-2 and LNCaP-SSR are more susceptible to growth inhibitory effects of the GSK-3β inhibitor SB21676 than LNCaP cells or the AR-negative PC3 cells

**Figure 7:**
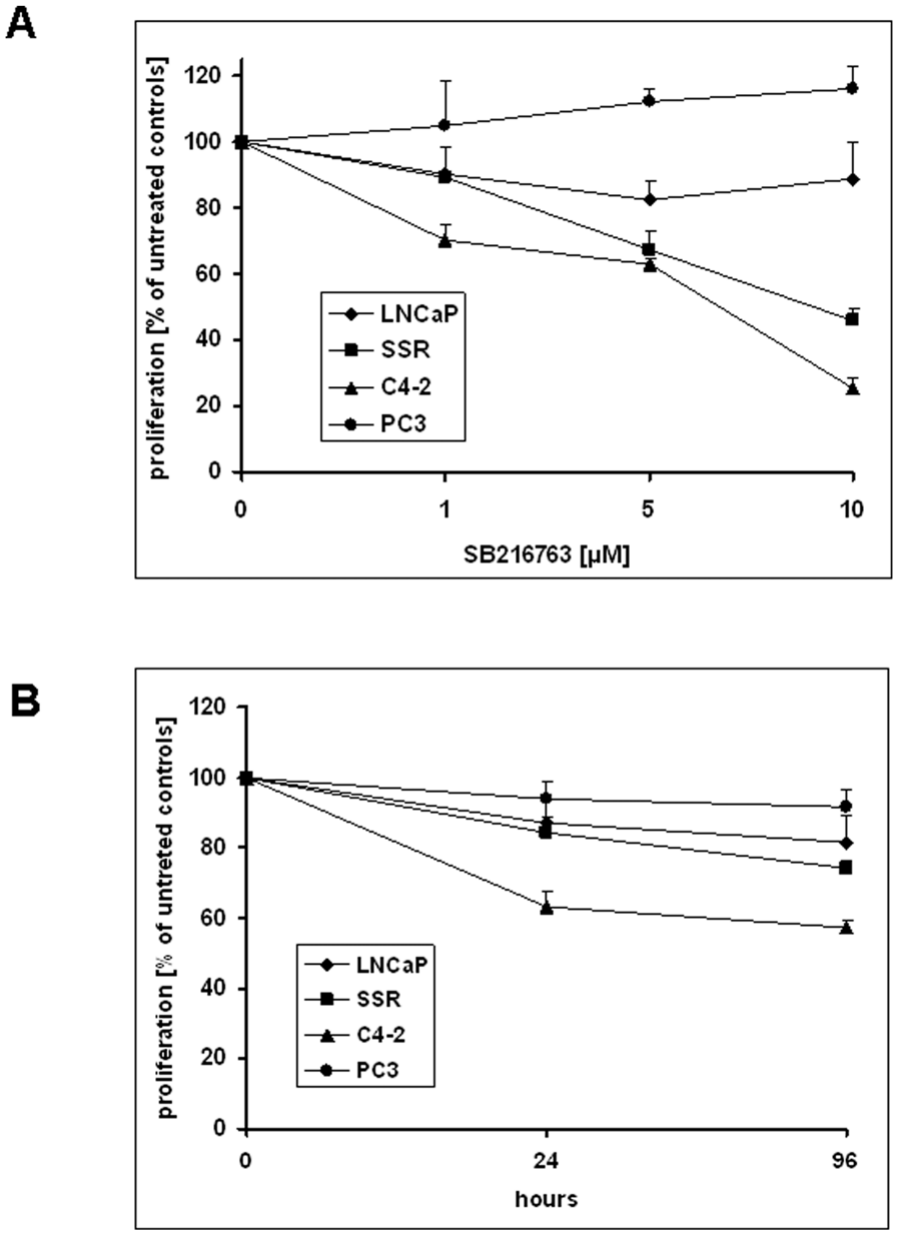
Inhibition of GSK-3β by SB216763 inhibits the proliferation in CRPC cells. AR-positive LNCaP, LNCaP-SSR and C4-2 cells as well as the AR-negative PC3 cells, were treated with increasing amounts of SB216763 for 48 hours (A) or with/without 1 µM SB216763 for 0, 24 and 96 hours (B). Proliferation was measured using an MTT assay as described in Material and Methods. Results shown under (A) are expressed as % of untreated controls ± standard deviation. Results shown under (B) are expressed in percent of SB216763-treated/untreated control which was set at 100% for the time point zero ± standard deviation.

Inhibition of GSK-3β has been shown to inhibit proliferation of AR-positive cells grown in presence of physiological levels of androgens [27], [32]. With a focus on CRPC cells, we examined the effects of SB216763 on the proliferation of PC cell lines grown under androgen-deprived conditions. As seen in Figure 7A, AR-positive CRPC cells grown in the absence of androgens were more susceptible to the growth inhibitory effects of SB216763 (C4-2>LNCaP-SSR>LNCaP). In a time course experiment (Fig. 7B), proliferation of the AR-positive CRPC cell lines LNCaP-SSR and C4-2 grown for 96 h in the presence of 1 µM SB216763 was inhibited by 26% and 35% respectively. In contrast, the proliferation rate of the parental LNCaP and the AR-negative PC3 was only reduced by 19% and 8% when cultured under the same conditions. In summary, the AR-positive LNCaP cells were more susceptible to SB216763 treatment than the AR-negative PC3 cells. The effect of SB216763 on cellular proliferation of PC cells grown under androgen-deprived conditions was the most pronounced in AR-positive CRPC cells (C4-2>LNCaP-SSR>LNCaP>PC3) (Fig. 7A, B).

### Inhibition of GSK-3β regulates AR-signalling by modulating intracellular localisation of the AR *in vivo*

**Figure 8:**
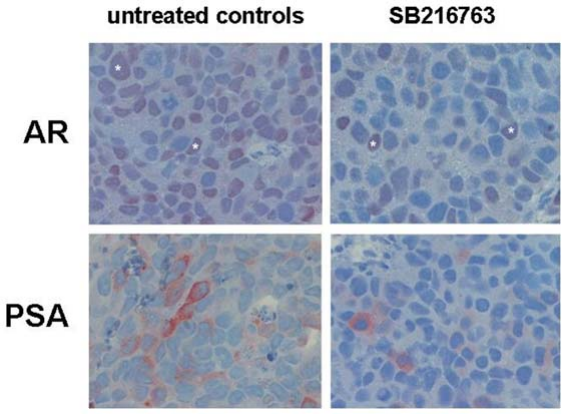
Inhibition of GSK-3β diminishes nuclear localization and function of the AR *in vivo.* The CAM assay was performed with C4-2 cells. Subsequently, nuclear localization of the AR and intracellular PSA distribution in untreated and SB216763-treated tumor-nodules (magnification 400x) was performed as described in Material and Methods. AR-positive cells are marked with stars.

In order to simulate the effects of a GSK-3β inhibition *in vivo*, we used a modified chick chorioallantoic membrane (CAM) model [25]. SB216763 was injected into a CAM-vein allowing the systemic spread of the compound in the chick embryo-CAM system. The effects of SB216763 on the engrafted tumor nodules were monitored after 48 hours by immunohistochemistry. In contrast to the *in vitro* observations not only C4-2 but also LNCaP expressed high levels of nuclear AR *in vivo* (nuclear AR: LNCaP: 74.6±25.8%, C4-2: 63.4±10%). Following SB216763 treatment nuclear AR levels in C4-2 cells were reduced by 57% from 63.3 to 27.1% after 48 hours (Fig. 8, Table 1). Nuclear AR levels in LNCaP remained nearly unaffected by SB216763 (LNCaP: untreated 74.6±25.8%; SB216763-treated 76.5±18%) (Table 3). The percentage of PSA-positive cells was higher in the C4-2 cell line as compared to the parental LNCaP cell line (% of cells staining for PSA: LNCaP 3±3.3% versus C4-2 16.8±10.6%), suggesting that the AR in C4-2 is active. Following SB216763 treatment the decrease of nuclear AR in C4-2 was paralleled by a decrease in PSA-positive cells (untreated C4-2: 16.8± 10.6% versus 3.8±4.7% in SB216763-treated C4-2) (Fig. 8, Table 3).

## Discussion

In western industrialized countries, PC presents a serious health problem and is the second leading cause of cancer deaths in elderly men. Although PC is very heterogeneous in its etiology, androgen signalling is a key element in the development and progression of PC. Initially, PC cells are largely dependent on androgens for growth and survival. As a consequence, endocrine therapy involving androgen depletion by surgical or medical castration, as well as the blockade of the androgen receptor with anti-androgens, has become a standard treatment for advanced or metastatic disease. However, the benefit from endocrine therapies of advanced PC is only transitory. Within a period of around 2 years, nearly all prostate cancers progress to a castration-resistant state of the disease where they do no longer respond to standard endocrine therapies. In the past, it has been hypothesized that the state of CRPC was due to a clonal selection of AR-negative cells. This assumption was mainly based on the Dunning rat tumor model where the development of an androgen-insensitive state is linked to the loss of the AR in tumor cells during androgen withdrawal. However, clinical studies showed that the AR is rarely lost but is often increased in CRPC tumor specimens and their metastases [5], [6], [34], [35]. The fact that in the absence of androgenic stimuli many of the same genes that are increased by androgens in androgen-dependent PC become elevated in CRPC supports the notion of constitutively active AR proteins in CRPC cells [36]. This hypothesis is also supported by the observation that a disruption of AR function inhibits proliferation of CRPC cells grown in the absence of androgens [37].

In this report we used the AR-positive LNCaP sublines C4-2 and LNCaP-SSR which are both known to grow and survive under androgen-deprived conditions as an *in vitro* tumor model for CRPC [21], [20]. We were able to show that, in contrast to the androgen-dependent parental LNCaP cells, castration-resistant LNCaP sublines C4-2 and LNCaR-SSR exhibited high levels of AR and PSA when grown in the absence of androgens. Most interestingly, the increase in intracellular AR protein levels was paralleled by an increase in GSK-3β, a ubiquitous serine threonine kinase shown to be an important modulator of AR stability *in vitro* [32], [27]. Although at the moment we are unable to say that the dysregulation of GSK-3β levels is responsible for the tendency of PC cells to increase intracellular AR-levels while becoming castration resistant, our *in vitro* observations are in line with a recent clinical study showing an accumulation of GSK-3β in cells of castration-resistant tumors [38]. The detection of high intracellular PSA levels in C4-2 and LNCaP-SSR devoid of androgenic stimuli suggests that the AR is functionally active in these cells even under androgen- deprived conditions.

In order to generate genomic signals, the AR has to be transported into the nucleus. In this study, the AR was found to be predominantly nuclear in LNCaP-SSR cells and C4-2 cells in the absence of androgens. The reason for the androgen-independent nuclear accumulation of full-length AR in CRPC cells remains largely unknown. Under normal conditions the nuclear translocation of the AR is dramatically increased upon hormone binding as demonstrated for LNCaP. Recent findings suggest that, in order to enter the nucleus, the AR has to undergo an intra-molecular conformation change that brings the N- and C-termini of the molecule into close proximity. This intra-molecular conformational change allowing activation and nuclear translocation of an AR monomer, prior to AR dimerization, is usually induced by ligand binding and only occurs in living cells, not in cell lysates [39], [40], which suggests that this intra-molecular reorganization of the AR is not protein-autonomous but depends on cellular factors. To test this hypothesis, we subsequently transfected C4-2 and LNCaP cells with an expression construct coding for a wild type AR fused to a green Eos fluorescent protein (EosFP) [41]. In the presence of DHT AR-EosFP was detectable in the nuclei of both LNCaP and C4-2. When grown in the absence of DHT, AR-EosFP was only detectable in the nuclei of castration-resistant C4-2 cells but not in the nuclei of androgen-dependent LNCaP cells. This observation is consistent with a similar experiment demonstrating a robust nuclear localization of a GFP-tagged AR transfected into C4-2 cells [42]. Taken together, these findings suggest that the nuclear localization of full-length AR in CRPC does not necessarily require hormone binding and may instead be regulated by other factors.

As recently shown by our group, short term inhibition of the serine/threonine kinase GSK-3β by small molecule inhibitors induced a rapid, CRM1-dependent nucleocytoplasmic shuttling of the AR in androgen-treated cells [19]. As GSK-3β is overexpressed in CRPC cells *in vivo and in vitro*, we hypothesized that the enzyme might also be part of a putative protein complex involved in the nuclear accumulation of the AR. This hypothesis is supported by a previous finding showing that GSK-3β is highly phosphorylated at tyrosine 216 (Y216), the activation function of the enzyme, in C4-2 cells [30]. The increase of GSK-3β^Y216^ phosphorylation in LNCaP cells following DHT treatment (Fig. 2) furthermore suggests an important role for GSK-3β in AR signalling under normal conditions. In an attempt to disrupt the predominant nuclear localization of the AR in CRPC cells, we treated castration-resistant C4-2 and LNCaP-SSR as well as the parental LNCaP with the maleimide SB216763. This highly potent compound is known to inhibit intramolecular tyrosine kinase activity of the GSK-3β molecule, the latter being responsible for its autophosphorylation at Y216 [33]. As seen in Figure 2, the GSK-3β inhibitor SB216763 is able to decrease Y216-phosphorylation of GSK-3β in LNCaP as well as in C4-2 cells. Following treatment with SB216763, the nuclear accumulation of the AR in the castration- resistant LNCaP sublines C4-2 and LNCaP-SSR was dramatically reduced in the presence, and most importantly in the absence, of DHT, being most pronounced in C4-2 cells (Table 2). Nucleocytoplasmic shuttling of the AR after GSK-3β-inhibition in C4-2 cells grown in the absence of DHT could be reversed by leptomycin B (LMB), suggesting a CRM1-dependent export mechanism (Fig. 5). In a previous study, we identified a CRM1 binding site in the C-terminus of the AR [19]. Due to its vicinity to the ligand binding domain, we hypothesized that hormone binding could regulate the accessibility of the CRM1 binding site, thereby modulating the nuclear export of the AR. The observation that the AR can be exported from the nuclei of CRPC cells growing in the absence of androgens is an important novel finding, clearly demonstrating that the nuclear export of the AR following GSK-3β inhibition is not due to a modulation of hormone binding properties of the receptor.

The fact that short term inhibition of GSK-3β (max. 4 hours) leads to a rapid export of the AR prompted us to analyze the effects of a long term inhibition of the enzyme in AR-positive CRPC-cells. Silencing of GSK-3β using specific shRNA led to a depletion of nuclear AR in C4-2 cells. Nuclear depletion of AR protein was less pronounced in C4-2 cells grown in the presence of the AR-stabilizing hormone DHT. Although these findings support the assumption that inhibition of GSK-3β triggers the nucleocytoplasmic shuttling of the AR, the data derived from the long term shRNA-experiments must be interpreted carefully. Short term inhibition of GSK-3β activity by small molecules like SB216763 was repeatedly shown to induce a rapid, CRM1-dependent nuclear export of the AR in PC cells [19], [32]. In contrast to these findings, the long-term inhibition of GSK-3β caused a proteasomal degradation of the AR protein [32]. Because GSK-3β triggers AR localization as well as AR stability, we hypothesize that the dramatic depletion of nuclear AR in C4-2 cells following GSK-3β silencing is due to a combination of nuclear export and proteasomal degradation of the receptor molecule.

The fact that inhibition of GSK-3β affects AR function as well as AR stability prompted us to analyze the effects of a GSK-3β inhibition on the proliferation of PC cells *in vitro*. Response to treatment of PC cells with SB216763 was most pronounced in AR-positive CRPC cells (inhibition of cell growth: C4-2>LNCaP-SSR>LNCaP>PC3). The ability of SB216763 to inhibit cellular proliferation was paralleled by its ability to induce a CRM1-dependent export of the AR in PC cells.

In order to test the efficacy of GSK-3β inhibitors as potential therapeutic agents, we extended our studies to a CAM-xenograft model. To our surprise the AR was predominantly nuclear in LNCaP xenografts as well as in C4-2 xenografts. A possible explanation for this could be the production of androgens by the host organism. Moreover, the AR of LNCaP and its derivatives is carrying a point mutation (T877A) leading to a promiscuous AR that can, at least in part, be activated by various non-androgenic steroids [43], [44]. Nevertheless, the AR of C4-2 as compared to the AR of the parental LNCaP is more active in the CAM-Xenograft model, as documented by intracellular PSA levels (cells positive for PSA: LNCaP 3,1% and C4-2 16.8%). Following a 48-hour treatment with SB216763, nuclear AR levels of C4-2 cells were significantly reduced, which was paralleled by a considerable reduction in intracellular PSA levels. In contrast to results in C4-2 cells the effects on AR and PSA in LNCaP cells remained insignificant.

Taken together our study demonstrates that (1) overexpression of the AR in CPRC cells is paralleled by an increase in intracellular GSK-3β, (2) the effects of GSK-3β on AR signalling are not due to a modulation of DHT binding to the AR, (3) nuclear accumulation of the AR in CRPC cells is not an autonomous function of a mutated AR receptor but is mediated by deregulated cellular factors, such as GSK-3β, (4) GSK-3β and CRM1 are part of a putative complex controlling the nuclear localization of the AR in CRPC cells, and (5) inhibition of GSK-3β activity by small molecule inhibitors induces a rapid nuclear export of the AR in CRPC cells *in vitro* and *in vivo* thereby modulating AR signalling.

The dependence of CRPC on transcriptionally active AR has resurged the interest in developing inhibitors targeting the AR or androgen axis. Next generation hormone therapies recognize the fact that in CRPC the AR can be activated by intrinsic production of tissue androgens as well as by peptide growth factors. Among hormonal agents currently being tested are CYP17 inhibitors like abiraterone, TAK-700 and TOK-001 or the second generation anti-androgen MDV-3100 [45]. Small molecule inhibitors like EPI-001 and sintokamide targeting the transactivation domain at the N-terminus of the AR have shown encouraging results in vitro as well as in experimental animal models [46], [47]. Our results suggest that simultaneous targeting of AR nuclear export as well as AR-stability by GSK-3β inhibitors is a further valuable strategy to diminish AR signalling in CRPC. Moreover, the combination of GSK-3β inhibitors with novel AR inhibitors might be a useful approach for the treatment of advanced CRPC.

## Materials and Methods

### Chemicals

GSK-3β inhibitor SB216763 (3-(2, 4-dichlorophenyl)-4-(1-methyl-1H-indol-3-yl)-1H-pyrrole-2,5-dione) was provided by Biomol GmbH, Hamburg, Germany. Phosphatase inhibitor cocktail tablets (Phos-Stop), protease inhibitor cocktail tablets (Complete Mini) and dithiothreitol (DTT) were obtained from Roche Diagnostics GmbH, Mannheim, Germany. Leptomycin B was a product of Alexis Biochemicals, Lörrach, Germany. Dihydrotestosterone (DHT), HEPES, ethylene-diamine-tetra-acetic acid (EDTA) and Tween 20 were provided by Sigma-Aldrich GmbH, Taufkirchen, Germany. Bovine serum albumin (BSA) was provided by PAA Laboratories GmbH, Pasching, Austria. Sodium dodecyl sulphate (SDS), Nonidet NP40 (NP40) and Glycine were products of AppliChem GmbH, Mannheim, Germany. All other chemicals, if not specified, were purchased from Sigma-Aldrich GmbH, Taufkirchen, Germany. GSK-3β-directed shRNA (pkD-GSK-3β-v1) or control shRNA (pkD-NegCon-v1) were products of Upstate Biotechnology, Lake Placid, NY.

### Antibodies

Antibodies for Western blotting: AR rabbit monoclonal antibody (Epitomics, Clone EP670Y) directed against the C-terminal end of the AR was purchased from Biomol, Hamburg, Germany. Polyclonal rabbit antibodies to pGSK-3β (Y216) and Lamin A (H102) as well as mouse monoclonal antibodies to PSA (CHYH2) and GSK-3β (1F7) were provided by Santa Cruz Biotechnology, Heidelberg, Germany. Mouse monoclonal antibody AC-15 to β-actin was purchased from Sigma Aldrich GmbH, Taufkirchen, Germany. Anti pGSK-3β (Ser 9) (D3A4) and anti-mouse and anti-rabbit IgG-horseradish peroxidase linked antibodies were products of Cell Signalling Technology, Frankfurt a.M., Germany.

Antibodies for histochemistry: Mouse monoclonal antibodies directed against PSA (ER-PR8) and AR (AR-441) were products of DAKO Diagnostica, Hamburg, Germany. Immunohistochemical detection/staining of primary antibodies was performed using Histostain Plus, Invitrogen, Darmstadt, Germany, according to the manufactures instructions.

### Cell culture

AR-negative PC3 cells and AR-positive LNCaP cells were provided by the American Type Culture Collection, Manassas, VA, USA. CRPC cell lines C4-2 and LNCaP-SSR were provided by Prof. Sven Reske, Ulm, Germany and Prof. Martin Burchardt, Greifswald, Germany. RPMI-1640, phosphate buffered saline (PBS) and penicillin/streptomycin solution were products of PAA Laboratories, Linz, Austria. Fetal bovine serum (FBS) and steroid-free dextran-charcoal-treated FBS (FBSdcc) were obtained from BioWest, Nuaille, France. Cell culture plastic ware was purchased from Sarstedt, Nürmbrecht, Germany. LNCaP cells and PC3 cells were routinely cultured in RPMI-1640, 1% penicillin/streptomycin (v/v), 10% FBS (v/v) whereas LNCaP-SSR and C4-2 were routinely grown in RPMI-1640 supplemented with 1% penicillin/streptomycin (v/v) and 10% steroid-free dextran charcoal-treated FBS (FBSdcc) (v/v). During experiments, cells were maintained in RPMI-1640 with 2.5% FBSdcc (v/v) and antibiotics in the presence/absence of DHT and the GSK-3β-inhibitor SB216763.

### Preparation of whole or nuclear cell extracts

For the preparation of whole cell extracts, cells grown in T25 flasks were incubated with 500 µl lysis buffer (50 mM Tris, 5 mM EDTA, 150 mM NaCl, 1% triton X 100 (v/v), pH 7.3) supplemented with phosphatase and protease inhibitors according to the manufactures instructions. Subsequently, cells were scraped thoroughly out of the flask. Cell lysates were centrifuged at 15000 xg for 15 minutes at 4°C in a tabletop centrifuge. Supernatants were collected and protein concentrations were determined using the BCA-Protein Assay (Pierce, Rockford, IL, USA). Whole cell extracts were subjected to Western blot analysis.

Nuclear extracts were prepared as recently described [19]. Briefly, cells grown in T25 flasks were lysed in 500 µl buffer A (10 mM HEPES, 1.5 mM MgCl_2_, 10 mM KCl, 0.5 mM DTT, 0.05% NP40, pH 7.9) and centrifuged 10 minutes at 3000xg to collect cytoplasmic fractions. Residual pellets containing the nuclei were resuspended in 93.5 µl buffer B (5 mM HEPES, 1.5 mM MgCl_2_, 0.2 mM EDTA, 0.5 mM DTT, 26% glycerol (v/v), pH 7.9) and 6.5 µl of 4.6 M NaCl and centrifuged at 15000xg to remove nuclear debris. Extracts were analysed by Western blotting.

### Western blot analysis and immunodetection

Whole cell extracts or nuclear fractions (30 µg) were subjected to electrophoresis in a 10% SDS-PAGE and electroblotted onto nitrocellulose membranes (Pall, Bad Kreuznach, Germany).

AR, PSA and GSK-3β were detected as follows: Non-specific binding sites on the nitrocellulose were blocked by incubation with 5% (w/v) non-fat dry milk (Sucofin, TSI GmbH, Zeven, Germany) in PBST (PBS + 0,1% Tween20). Antibodies were applied in PBST supplemented with 1% not-fat dry milk as follows: AR antibody (EP670Y) 1∶2000; PSA antibody (CHYH2) 1∶500; GSK-3β antibody (1F70) 1∶500 and antibodies serving as loading controls for cytoplasm/nucleus, i.e., β-Actin antibody (AC15) 1∶20000 and Lamin A antibody (H102) 1∶1000. Resulting immunocomplexes were detected using horseradish peroxidase-labelled goat anti-rabbit antibody 1∶2000 and goat anti-mouse antibody 1∶2000 (1∶5000 for the detection of AC-15 to β-Actin). Immunoreactive bands were visualized by direct detection with Super Signal West Pico and Dura Chemiluminescence Substrates (Pierce, Rockford, IL, USA) using ChemiSmart 500 Vilber Lormat (Marne-la-Valle, France). Immunoreactive bands were quantified by densitometry using ChemiCapt/Bio1D software from Vilber Lormat.

Immunodetection of site specific phosphorylation in GSK-3β (pGSK-3β^Y216^ or pGSK-3β^S9^) was performed using the following modifications: Nitrocellulose membranes were blocked in PBST supplemented with 5% BSA. Subsequently membranes were incubated with a phosphospecific antibody to pGSK-3β (Y216) diluted in PBST with 1%BSA (w/v) at 1∶500. Immunoreactive bands were detected and visualized as described above. After detection of pGSK-3β^Y216^ the phosphospecific antibody was removed by incubating the membrane 2×10 minutes in stripping buffer (1.5% Glycin (w/v), 0,1% SDS (w/v), 1%Tween-20 (v/v), pH 2,2). Following three washing steps (3×10 min) in PBST, the nitrocellulose membrane was blocked in PBST supplemented with 5% BSA and reprobed with the pGSK-3β (S9) antibody (1∶1000). Subsequently, the membrane was stripped once again and reprobed with the GSK-3β antibody (1F7) (1∶500), serving as a loading control. Immunoreactive GSK-3β bands were detected and quantified as described above.

### Nuclear translocation assay

Nuclear translocation of the AR was analyzed in LNCaP and C4-2 cells transfected with pAR-t1EosFP, an expression plasmid coding for a green fluorescent AR-Eos fusion protein (AR-EosFP) [22]. Twenty four hours after transfection cells were treated with/without SB216763 in the presence/absence of DHT [19]. Subsequently, fluorescent cells were analyzed by fluorescence microscopy.

### Silencing of GSK-3β in prostate cancer cells C4-2 cells

C4-2 cells were transiently transfected with shRNA-expression plasmids directed against GSK-3β (pkD-GSK-3β-v1) or with control shRNA (pkD-NegCon-v1) as recently described [32]. High transfection efficiency was achieved in the LNCaP C4-2 subline using Attractene Transfection Reagent, Qiagen, Hilden, Germany.

### Cell viability assay

Cell viability was determined by means of a colorimetric MTT-assay measuring the reduction of tetrazolium salts to formazan derivatives by functional mitochondria. The assay was performed as originally described [23].

### Chorioallantoic membrane assay (CAM Assay)

In order to analyze the effects of a GSK-3β inhibition *in vivo*, we used a chick chorioallantoic membrane (CAM) assay as an animal substitute model [24]. In this assay, shells of fertilized chicken eggs were opened on day 8 and silicon rings (5 mm in diameter) were applied onto the CAM. 10^6^ cells were seeded into the ring in 20 µl 50% Matrigel (v/v) (BD Biosciences, Heidelberg, Germany) dissolved in serum and antibiotic-free RPMI-1640. Tumor grafts were allowed to grow for 48 hours. In contrast to the classical CAM assay, the GSK-3β inhibitor SB216763 was not applied topically. Instead 50 µl of the GSK-3β inhibitor [normal saline, 2% Dimethylsulfoxide (v/v), 100 µM SB216763] were injected into a CAM vein [25], allowing the systemic spread of the compound in the chick embryo CAM system for 48 hours. Subsequently, tumor tissues were fixed, paraffin embedded, and serially sectioned. The effects of SB216763 on AR and PSA in the engrafted tumor nodules were monitored by immunohistochemistry (antibodies to AR, (AR-441) 1∶500; and to PSA (ER-PR8) 1∶25) as previously described [24], [25].

### Statistical Analysis

The Mann-Whitney U test was used to evaluate distribution difference across two groups. Statistical significance in this study was set as p≤0.05.

## Supporting Information

Figure S1 **Phosphorylation of the GSK-3β downstream target β-catenin.** AR-positive C4-2 cells were treated with increasing amounts of SB216763 for 24 hours in the presence/absence of DHT. Cell extracts were analyzed by Western blotting as described in Material and Methods. Phosphorylated forms of β-catenin were determined using a rabbit polyclonal Phospho-β-Catenin(Ser33/37/Thr41) antibody (Cell Signaling Technology, New England Biolabs, Frankfurt a.M., Germany). Subsequently, membranes were stripped and reprobed with an antibody directed against native β-catenin (rabbit monoclonal antibody, Epitomics) which served as a control.
(TIF)
