# Supplementary figures and images for: Inhibition of Glycogen Synthase Kinase-3β Counteracts Ligand-Independent Activity of the Androgen Receptor in Castration Resistant Prostate Cancer
Source: PLoS One. 2011 Sep 29;6(9):e25341. doi: 10.1371/journal.pone.0025341 (PMC3183056; doi:10.1371/journal.pone.0025341)

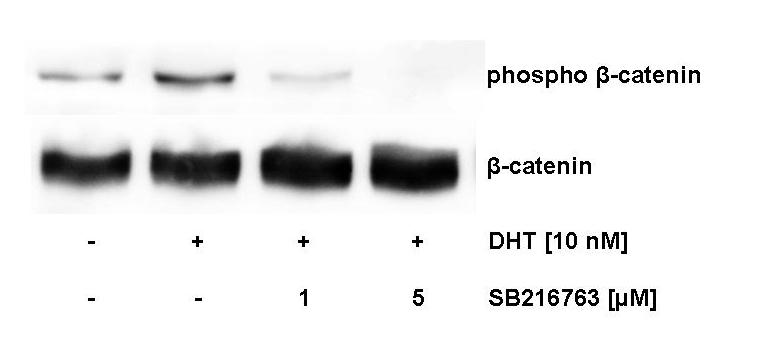

Supplement: Figure S1 — Phosphorylation of the GSK-3β downstream target β-catenin. AR-positive C4-2 cells were treated with increasing amounts of SB216763 for 24 hours in the presence/absence of DHT. Cell extracts were analyzed by Western blotting as described in Material and Methods. Phosphorylated forms of β-catenin were determined using a rabbit polyclonal Phospho-β-Catenin(Ser33/37/Thr41) antibody (Cell Signaling Technology, New England Biolabs, Frankfurt a.M., Germany). Subsequently, membranes were stripped and reprobed with an antibody directed against native β-catenin (rabbit monoclonal antibody, Epitomics) which served as a control. (TIF) [file pone.0025341.s001.tif]
